# Supporting continuing bonds for parents with infants with uncertain futures on neonatal units in the United Kingdom: co-designing a culturally sensitive music therapy intervention

**DOI:** 10.3389/fpsyt.2025.1633878

**Published:** 2025-07-21

**Authors:** Kirsty Jane, Ruby Hayns-Worthington, Katie Gallagher, Polly Livermore, Helen Shoemark, Glenn Robert

**Affiliations:** ^1^ Methodologies Division, Florence Nightingale Faculty of Nursing, Midwifery & Palliative Care, King’s College, London, United Kingdom; ^2^ Elizabeth Garrett Anderson (EGA) Institute for Women’s Health, University College, London, United Kingdom; ^3^ Great Ormond Street Hospital NHS Trust, Zayad Centre for Research, London, United Kingdom; ^4^ Boyer College of Music and Dance, Temple University, Philadelphia, PA, United States

**Keywords:** experienced-based co-design, music therapy, cultural sensitivity, neonatal intensive care, neonatal palliative care

## Abstract

**Introduction:**

Neonatal intensive care is a traumatic environment for parents, infants and staff. Services need to collaborate with their users to develop acceptable and sustainable support. Music therapy can have positive impacts on physiological and psychological outcomes for infants and has the potential to help create a supportive service environment. This study aimed to co-design a culturally sensitive music therapy intervention to support the development of continuing bonds for parents with infants with uncertain futures on neonatal units.

**Methods:**

An Experience-based Co-design approach was implemented to identify areas of challenge within neonatal units before designing solutions informed by a music therapy approach. Across four co-design meetings, six parents, five staff and three charity representatives worked collaboratively and co-analysed qualitative data to develop a culturally sensitive music therapy intervention.

**Results:**

Three themes were identified from the data: trauma, identity and staff-parent relationships. A logic model was constructed which guided the intervention development, leading to a trauma-informed intervention comprising a) a musical gift created by external family and friends supported by a music therapist b) parent and staff playlists c) a journal to guide use of music and encourage self-reflection and d) ‘ask me’ staff badges to start staff-parent conversations.

**Discussion:**

This is the first co-designed neonatal music therapy intervention. Through increasing social connections and promoting individuals' strengths, it has the potential to increase psychological safety for both parents and staff.

## 1 Background

Families expecting the birth of an infant will often have preconceptions of what their experience of birth and early days with their infant will be like. If their infant is admitted to a neonatal unit (NNU), these are quickly disrupted as they manage the stress of the admission on family life (1). Reasons for admission to the neonatal unit can include the infant’s need for specialist medical care due to prematurity as well as for multiple physiological conditions. Often admission is unexpected, as the majority of infants are born at full term (>37 weeks gestation) (2). In the UK, the average length of stay in NNU can range between 4–92 days (3). Being in the neonatal unit can be a major interruption to the natural parent-infant bonding process and is often distressing for parents (4).

The neonatal unit is a particularly stressful environment for parents, staff and infants. For infants, they experience separation from their parents, painful procedures and life in a medicalised environment which increases their risk of having psychosocial difficulties as they grow up (5, 6). Many parents may experience repeated traumatic events through witnessing the emergency care of other infants as well as the fragility of their own infant’s life (7). Often infants may need to be transferred to another unit at some point in their care due changes in their medical needs, which leads to further stress for parents (8). Even post-stabilisation, many parents find that the prognosis of their infants’ future remains uncertain, with many families meeting the requirements for receiving palliative care support (9). Due to increased success in stabilising and supporting extremely premature newborns and those with congenital malformations there has been an increase in the prevalence of children with uncertain futures under the age of 1 (10, 11). Many of these infants will experience prolonged life in intensive care and possibly die before experiencing time outside of the unit. There are known mental health implications for parents on neonatal units, with some parents experiencing symptoms of Post-Traumatic Stress Disorder (PTSD) (12, 13). Such factors are known to impact the parent-infant relationship and require support to improve outcomes (14). Equally, research suggests that staff experience moderate to high levels of secondary traumatic stress and burnout on neonatal units (15, 16). Such levels of stress can negatively impact staff’s ongoing relationship with parents and reduce their ability to support parents emotionally (17) leading to a negative impact on infant care (18).

Many parents and professionals will also experience discrimination and abuse due to the intersections of identities including socioeconomic status (19–21). Additionally, many will experience physical and emotional harm caused by psychological reactions resulting from historical oppression and marginalisation that can transcend generations (22). These factors result in social disadvantage impacting healthcare professionals and parents neonatal experience and can lead to major disparities in health outcomes (23). This phenomena has been reported globally (24). Health care systems that do not acknowledge and respond to such social and historic trauma are likely to perpetuate or increase the trauma symptoms of service users (25). In the UK, for example, oppression as a result of white supremacy perpetuates a lack of culturally, linguistically and religiously appropriate healthcare which contributes to continued health inequalities in neonatal care, with greater infant and maternal mortality occurring in Black and Asian families (26).

Increasingly, Music Therapy (MT) is being considered by neonatal healthcare leads for wider implementation within healthcare services (27). Research confirms that MT can have both positive psychological and physiological benefits for infants, improving brain function, sleep and feeding (28–30). In addition, MT is thought to support maternal mental health through providing opportunities for relaxation and connection with their infant (31). Studies also suggest that it can reduce parental state anxiety (32). A recent systematic review found evidence to suggest that MT interventions can enhance the mental health of healthcare professionals by reducing symptoms of stress, anxiety and burnout (33). However, there have been challenges to implementing MT within units, with some reports of parental embarrassment and anxiety resulting from the expectation that they will participate in therapy (34, 35). Additionally, infants with an uncertain future are often excluded from neonatal music therapy research resulting in the appropriateness of current approaches remaining unknown (36). Some qualitative studies have reported largely positive staff attitudes to MT in adolescence services and children’s mental health settings (37, 38). While some studies have also canvassed the views of staff in neonatal units (39, 40), little is known about how supportive staff in the UK are about the integration of MT into standardised neonatal service provision.

Although specialised training for approaches to MT on the NNU are available globally and acknowledged (41, 42), it is not known how UK neonatal professionals view the value and effectiveness of neonatal MT practices. Despite the potential of MT to support the wellbeing of parents, infants and staff, there is a lack of standardised practice of MT on NNUs currently in the UK. This study therefore aimed to co-design a MT intervention to support parental bonding with infants in the NNU with uncertain futures. Creation of an intervention that focused on acceptability for those with infants with uncertain futures is a priority due to the likelihood of many families experiencing this sense of uncertainty during admission (even though many may leave the unit without complex care requirements). It is hoped that through this approach the acceptability of MT to neonatal families would be increased and the intervention be appropriate to all neonatal families. It was paramount that the intervention was culturally appropriate for the diverse range of families whose infants are admitted to NNU. The research also aimed to develop an intervention which would be both acceptable and accessible to a diverse range of parents and professional staff in this setting.

Design based approaches are increasingly being used to encourage stakeholder collaboration for the purposes of improving healthcare services (43). Experience-Based Co-Design (EBCD) is one approach that promotes joint working with staff and service users to improve clinical care and healthcare environments (44). EBCD has been used effectively to enhance patient care and experiences (45, 46). Recently this has included the collaborative development of resources which support parents in their roles as caregivers of children with medical requirements, such as children with cerebral palsy (47) or those with rheumatology conditions (48). Such participatory approaches can also be used to consider the acceptability of new interventions to stakeholders and can improve their chances of implementation and sustainability within healthcare systems (49).

The main aim of this article is to provide a detailed description of the co-design process of a culturally sensitive MT intervention to support the continuing bonds of parents with infants with uncertain futures on NNU.

## 2 Method

### 2.1 Research team

The lead researcher (KJ) is a specialist neonatal music therapist with expertise in neonatal palliative care. In their role as a music therapist on a level 3 NICU and at Noah’s Ark Children’s Hospice, they had worked directly with some parent members of the co-design team as a music therapist while their infant was in neonatal care. However, at the time of the study KJ no longer had a therapeutic relationship with any member of the group. Additionally, some co-design members who were healthcare professionals had worked with the lead researcher professionally in neonatal care. A team approach ensured that any potential bias from prior relationships was minimised. The wider research team (KG, PL, HS, GR) had a range of clinical expertise in MT, neonatal and child health care as well as research expertise in service development and co-design but had not worked clinically with the lead researcher or co-design members. All researchers identify as White.

### 2.2 Ethical approval

The study design and procedures were approved by NHS Health Research Authority Research and Ethics Committee June 2024. REC reference: 24/NW/0113. IRAS project 335663. Written consent was taken before any participation in the co-design process.

### 2.3 EBCD adapted approach

**Figure 1:**
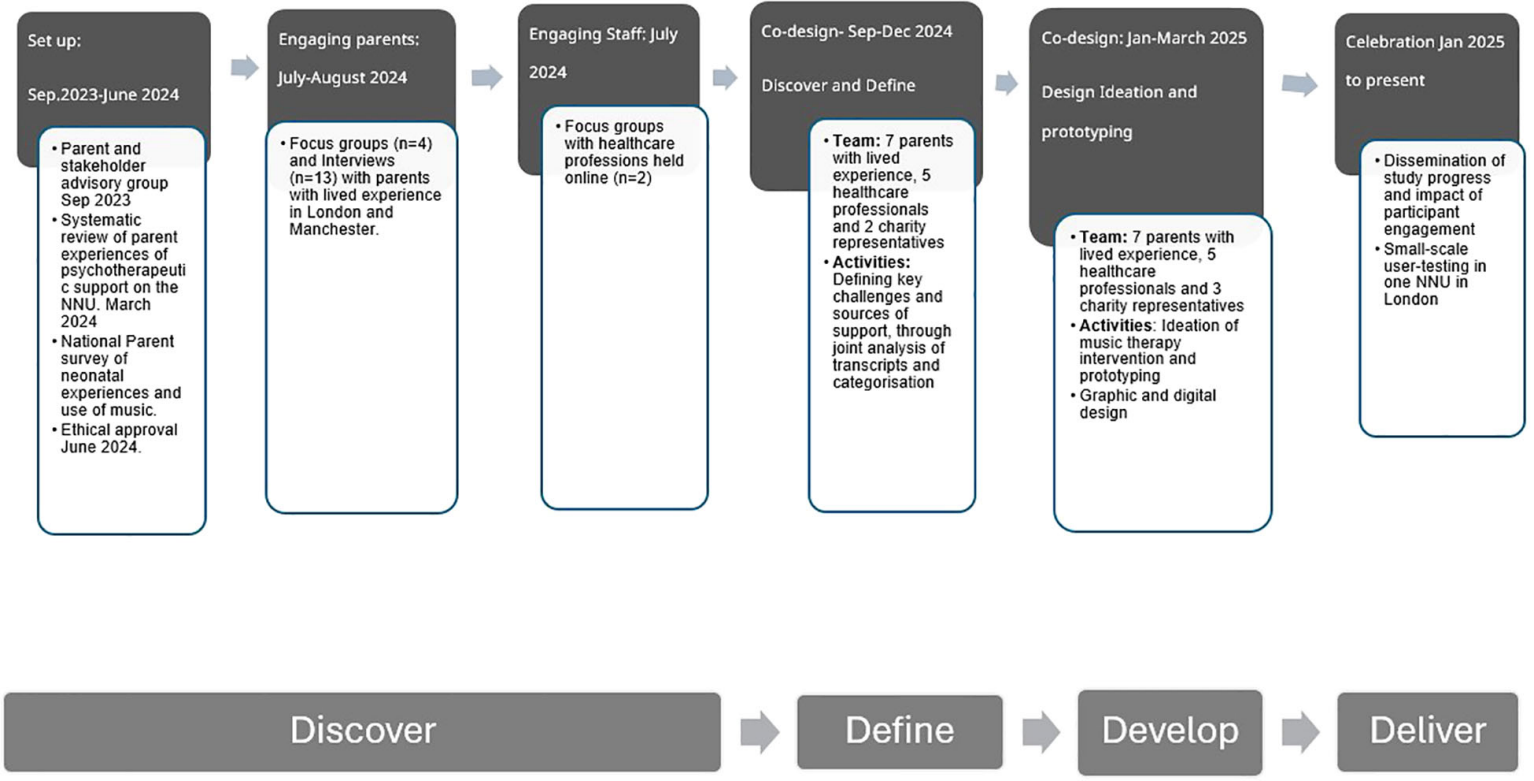
Experience-based co-design process.

EBCD is traditionally a six-stage process consisting of iterative rounds of data collection and analysis as well as co-design (50). This study used an adapted form of EBCD as underpinned by the ‘Double Diamond’ design process approach which consists of four stages: Discover, Define, Develop, and Deliver (Design Council, 2019) (see **Figure 1**). This project ‘discovered’ and ‘defined’ the challenges of neonatal parents and staff before collaboratively ‘developing’ a MT intervention suitable to be ‘delivered’ in the neonatal environment. A triangulation of data from multiple sources was conducted to increase the validity of the final design. This included a systematic review (51), a national survey (52) and focus groups and interviews held in different geographical locations to increase the diversity of participant engagement (53), as well as regular member checking (54). Additionally, the collaborative approach of EBCD supports an analysis process which enables the prioritisation of the values and opinions of those with lived experience, thereby reducing researcher bias.

The ‘discover’ phase was conducted in Manchester, London and online from June 2023 - July 2024 and uncovered the multiple experiences of trauma endured by parents and staff on neonatal units as well as the loss and suspension of their identity. Additionally, it highlighted the impact that the infant, parent and staff had on each other and the value of strengthening the parent-staff relationship to improve neonatal experiences across the whole system. This phase was carried out collaboratively with parents and staff with lived experience to bring the voices of parents and staff with lived experience nationally to the fore. Detailed findings from the ‘discover’ phase are presented in separate papers (51–53).

**Figure 2:**
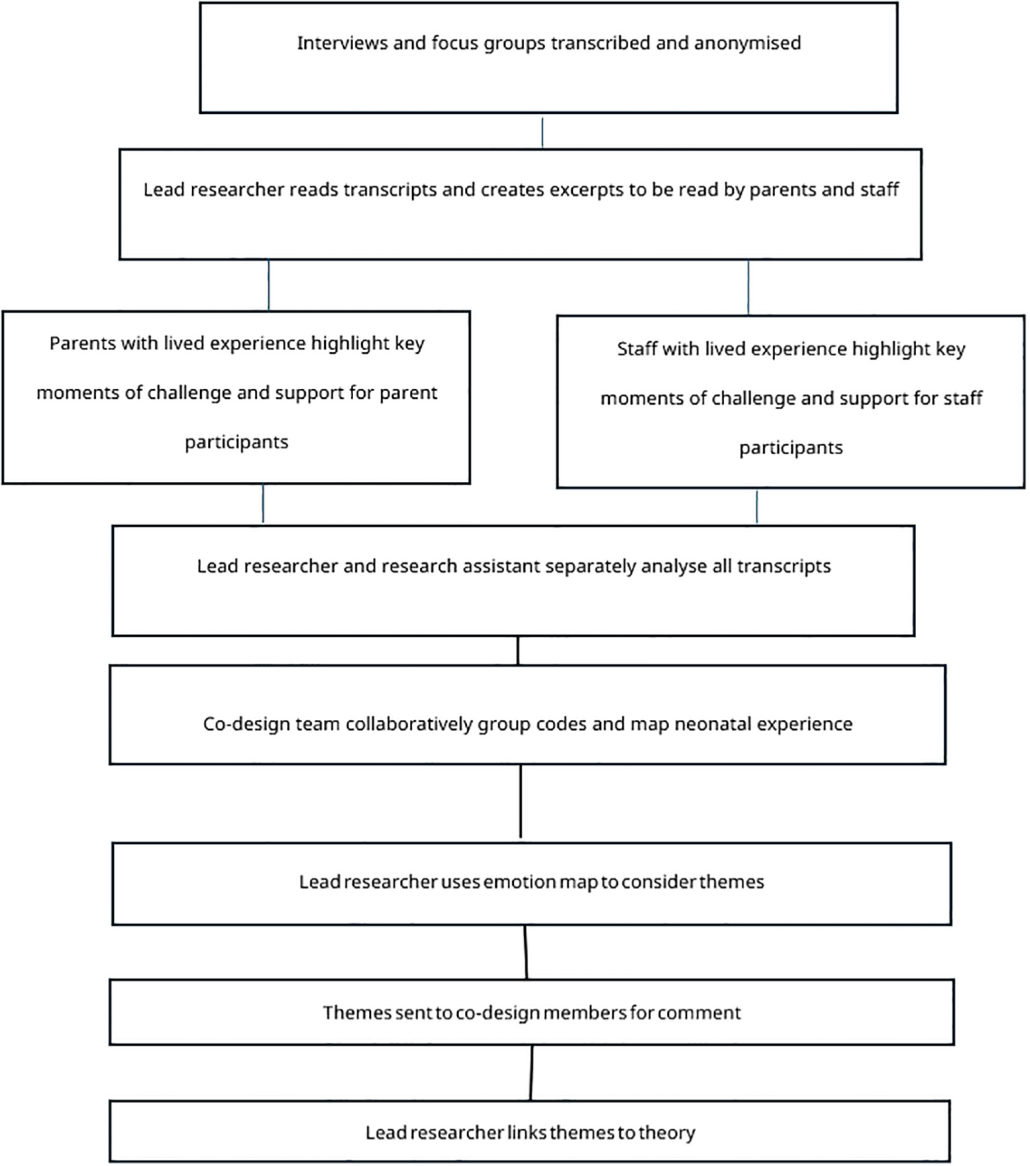
Flow chart of the data analysis process.

The ‘define’ phase of the process lasted six months (September to December 2024) and analysed data collected in the previous ‘discover’ phase which comprised a national survey, interviews and focus groups. Defining the ‘problem’ that the intervention would aim to address was carried out in the first two of the four co-design meetings. This analysis was conducted collaboratively over two meetings and included the collaborative coding of transcripts from the focus groups and interviews with co-design participants and then the clustering of these codes in combination with codes from the survey which were then mapped and used to define themes. After the second meeting the lead researcher (KJ) used themes that had emerged from this analysis to consider which theoretical lenses would be most suitable to interpret the data and to inform the development of the intervention in the third and fourth co-design meetings. A flow chart of this data analysis process can be found in **Figure 2**.

The ‘solution’ was developed in co-design meetings 3 and 4. Firstly, an intervention was created through the generation of ideas in meeting three which were then refined by the lead researcher working with a design team to create a digital visualisation of the intervention. This was presented in meeting four to co-design participants for further discussion and refinement.

## 3 Co-design participant recruitment

Parents with lived experience of having an infant with an uncertain future on the neonatal unit who i) either continued to require complex medical care or ii) were since bereaved were eligible to participate in all phases of the study. An initial open invite was made by the lead researcher to parents accessing support through Noah’s Ark children’s hospice where the lead researcher works, and charity representatives. These charities included PEEPs-HIE who support families who have experienced a hypoxic event, Camp Simcha a charity providing support for Jewish families with children requiring complex care and Across Ummah, a community support charity working closely with families in Manchester to address health inequality and improve living conditions of people in their community. The aim was to develop an initial advisory group that would inform all aspects of the study design and accessibility. Recruitment to this group was continued until representatives of multiple intersects of parental identities such as gender, languages spoken, ethnicity and religion and infant diagnosis was attained. These parents and charity leads were then offered the opportunity to continue as co-design members in collaboration with neonatal healthcare professionals across England who had approached the researcher with interest in developing music therapy or supporting the study after either witnessing their work with families on units or in presentations or seeing study advertisements. Charity representatives were included in the design group due to their close work with neonatal families. Collaboration with these charities was crucial to promote engagement with a wide population of neonatal parents and gain feedback from those wider than the co-design group. It was hoped that they would provide further representation of parent experience, be able to advise on the accessibility for the families they support and advocate for their community’s response to the study and intervention components. The characteristics of the co-design participants can be found in **Table 1**.

**Table 1:** Characteristics of co-design participants.

Participant	Gender	Age	Ethnicity	Participant type	Design activity	Co-design workshops attended			
						1	2	3	4
P1	Female	37	White Other	Bereaved Parent	D, A, I, P	x	x	x	x
P2	Male	46	White British	Bereaved Parent	D, A, I	x	x	x	
P3	Male	33	Asian Indian	Bereaved Parent	D, A, I,	x	x	x	
P4	Female	35	Asian Indian	Bereaved Parent	D, A, P	x	x		x
P5	Female	50	Asian Pakistani	Parent	D, A, I, P	x	x	x	x
P6	Female	45	Black African	Parent	D, A, P	x	x		x
P7	Female	39	White British	Parent	D, A, I, P	x	x	x	x
P8	Female	50	White British	Occupational therapist	A, I,	x	x	x	
P9	Female	51	White British	Occupational therapist	A, I, P	x	x	x	x
P10	Female	33	White Other	Consultant Neonatologist	A, I, P	x		x	x
P11	Female	67	White Irish	Nurse Educator	A, P	x	x		x
P12	Female	33	White British	Nurse	A, I, P	x	x	x	x
P13	Female	47	White British	Charity representative	D, A, P	x	x		x
P14	Male	37	White British	Charity representative	D, A, P	x	x		x
P15	Female	unknown	Black African	Charity representative	D, I, P			x	x

Key to ‘Design activity’: D, Study Design; A, Analysis; I, Ideation; P, Prototyping.

The lead researcher (KJ) led the four co-design meetings. All participants were provided with lunch and refreshments at each of the three in-person co-design meetings held at Noah’s Ark Children’s hospice (with the fourth meeting held online) and were reimbursed for their time and travel. The involvement and engagement of parents and HCPs was considered throughout and reported in this article in accordance with GRIPP2 reporting guidelines for involvement of patients and public in research (55).

As a result of the chosen recruitment strategy, five parent members had received MT with the lead researcher on the neonatal unit and three professionals witnessed MT provided by the lead researcher on their neonatal unit. To reduce participatory bias, a reflexive discussion was held at the end of each meeting to consider any internal challenges that were experienced by members when reading opinions and values that differed from their own, as well as to emotionally debrief. The lead researcher also maintained a reflexive journal throughout the study and had regular supervision. Finally, findings were shared on social media and in a newsletter with participants in the wider study to invite feedback at each stage.

## 4 The ‘define phase’.

### 4.1 Co-design meetings 1&2 (define phase)

The first meeting involved the lead researcher working with parent and staff groups analysing anonymised transcripts from focus groups and interviews from the ‘discover’ phase. Staff and parents met separately at this point to optimise safety of discussion and reflection without fear of judgement. The aim of these initial meetings was to define the key ‘touchpoints’ that the intervention could aim to improve and explore how MT might be used to achieve this. Touchpoints are key events that shape an experience either positively or negatively (56). Based on guidance from a previous study which involved people with lived experience in the qualitative analysis process (57), a selection of excerpts from transcripts were provided to maximise discussion but minimise data overload for participants. Given the often highly emotive content within the transcripts, it was hoped that this approach would also minimise the potential of secondary trauma. The lead researcher collated excerpts from the parent interview transcripts that represented the most common touchpoints, such as challenges with the involvement of others in the care of the infant and coping with the uncertainty of the future. Excerpts represented voices of participants of different genders, multiple ethnicities, and bereaved parents and those with children continuing to require complex care. They also contained reflections on the religious and secular use of music. At the request of the parents in the co-design groups, outlines of the content of the excerpts were provided ahead of the analysis meeting to allow parents to prepare for or opt out of reading them. However, no parents opted out of this phase on receipt of the content outlines.

**Figure 3:**
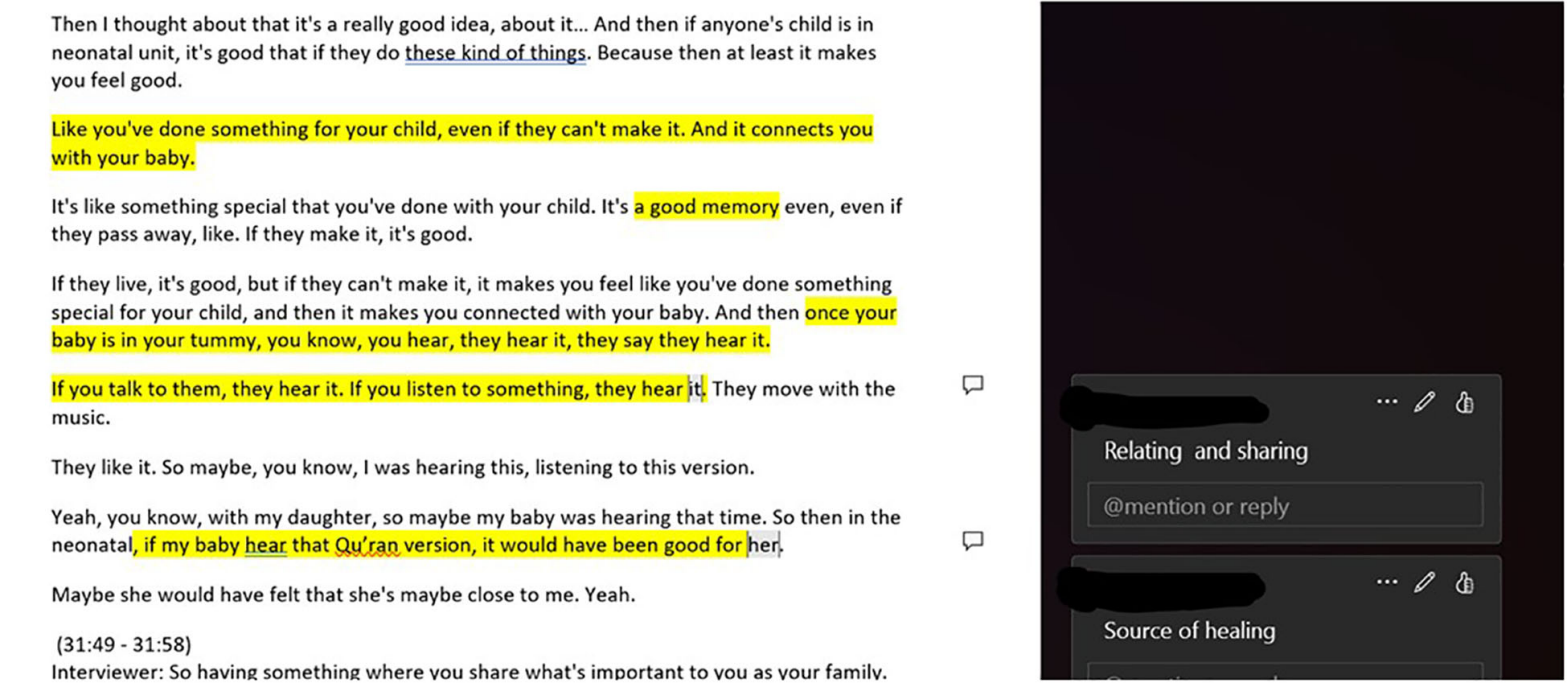
Digitalisation of parent group annotated transcript highlighting touchpoints for the participant’s use of music and reflection on the potential of music therapy.

The first hour of the first meeting was used to analyse parental neonatal experiences. Parents highlighted paper copies of the excerpts in colour and added their thoughts on the sections in the margins. These highlighted areas and notes where then discussed as a group and similarities identified across excepts read by each member. After a break, the next hour was used to consider interviewees use of music and the perceived effect of this music. A final discussion and reflection on the key touchpoints identified was held. After the meeting, the lead researcher transferred the annotated copies of the excerpts back into digital format for further analysis. An example of an annotated transcript can be found in **Figure 3**. A similar analytical process was conducted in a separate meeting with staff from the co-design group using excerpts of staff focus group transcripts which represented the unique challenges and support needed for staff on the NNU and their reflections on the potential of music therapy.

The lead researcher (KJ) and nurse researcher (RHW) then independently thematically analysed the full transcripts of all interviews and focus groups using NVivo software and *in vivo* coding (58) by highlighting key words or phrases used by participants. These *in vivo* codes were then jointly reviewed before being clustered into a secondary cycle of focused codes guided by the prior discussions and highlighted touchpoints from parents and staff. For example, the sight of the infant, watching procedures, machines beeping and smells of the hospital were clustered as ‘sensory based trauma’. These cluster codes were then taken to the second co-design meeting.

**Figure 4:**
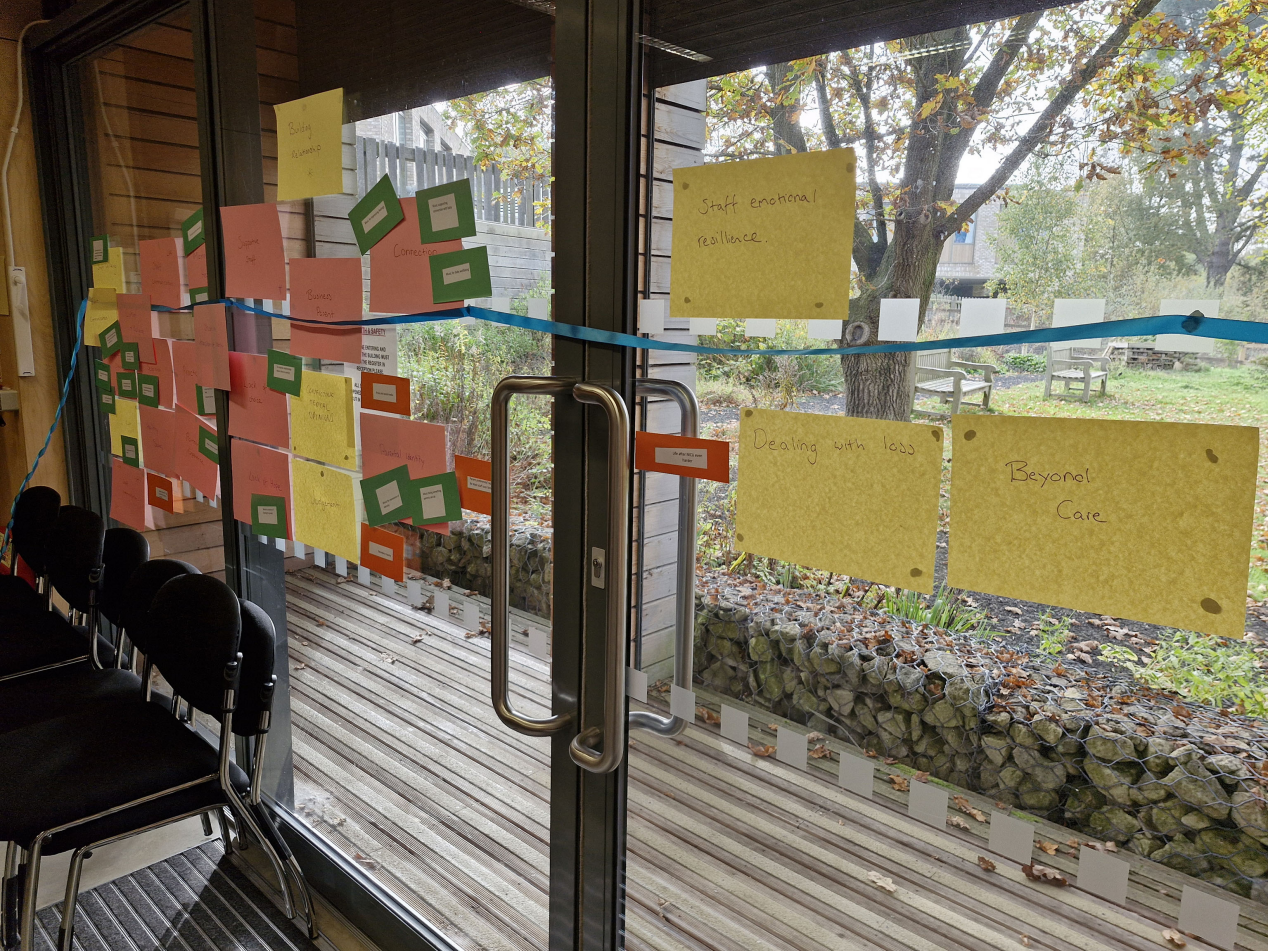
Emotion map of parent and staff neonatal experiences (A4 paper in two shades) with smaller cards overlaying musical challenges and support (smaller cards), all on timeline (Ribbon).

In the second 3-hour meeting, which included both parents and healthcare professionals, the co-design members were provided with the cluster codes and asked to group them into larger categories (see **Supplementary Material 1**). Collaboratively, the team read the parent cluster codes and grouped them, and then repeated the process with the staff codes, whilst discussing their decisions and interpretations. In the second part of the meeting the co-design team was asked to use these larger categories to create an emotion map (Point of Care Foundation, 2025) which visually presented parent and staff experiences from the beginning to end of the neonatal journey (see **Figure 4**). During this process, the team highlighted that many categories were events that could be experienced on multiple occasions. The group was therefore asked to place these events when they first occurred and mark the paper with a star to signify that it was a repeat experience. Codes of the potential and challenges for the use of music were then overlayed.

Throughout the joint meeting, the lead researcher facilitated the generation of conversation to deepen understanding of the groups and support each member to be heard. In situations where either parents or staff began to split or dominate conversation, the lead researcher invited insight from the other group to readdress the dynamics of the group. After the meeting had been held, the lead researcher wrote a newsletter which explained the process and findings of the analysis phase. This was distributed to all participants and charities who had engaged with the qualitative phase of the study to ensure that everyone who had supported or participated in the study was informed of study progress and their impact on the intervention development. It also invited participants the opportunity to comment further on findings at this stage.

### 4.2 Findings from the co-design meetings 1&2 (define phase)

#### 4.2.1 Thematic analysis

After meetings 1 and 2, the lead researcher created themes of participants’ neonatal experiences drawn by observing commonality in the clusters (see **Supplementary Material 1**). There were three primary themes which the lead researcher sent to the wider research and co-design team to review before being defined and agreed:

- The liminality of identities as (i) parents moved from being themselves to attempting to establish parental identity in the neonatal environment and (ii) staff suspended their own identities due to expectations of a professional persona.
- Trauma: which was experienced by both parents and staff through sensory experiences, secondary trauma and social trauma which had an impact on social relationships, sense of self, and emotional and physical regulation.
- Impact of staff-parent relationship: there was a clear impact that staff and parents had on one another, demonstrating the need to consider support holistically.

Analysis of participants use of music found that music was widely considered to have the potential to support both parent and infant wellbeing. It was also considered valuable to helping bridge relationships across cultural differences. In the discussions of the themes, most parents spoke about music in the form of ‘sound and song’ and of being something that was unique and personal to their family and identity. Whilst parents expressed wishes that they had been encouraged to engage with their infants through sounds and song, there were also concerns that a music therapist might ‘take away’ something that was deeply personal and that they themselves could do.

#### 4.2.2 Connecting findings to theory

To support the development of a logic model that would inform the development phases of the design process the lead researcher linked themes from the ‘define’ phase to relevant theory.

Theme 1: Identity

The uncovered problem of a sense of liminality of identity for parents was further explored through theories of liminality and the life course framework. The impact of uncertainty and helplessness was categorised by the co-design team as areas which maintained a period of liminality. Using a life course framework, Black et al. (2009) discovered that during periods of uncertainty parents often narrowed their social networks, which could also be seen in this study in the ‘loss of identity’ cluster in which participants reflected on feeling alone. Therefore, strengthening connections to external social connections and using music as the shared experience which connected those outside as well as enhancing connection with those on the unit became a focus of the intervention.

Staff suspension of identity was considered in two parts: firstly, because staff manage and limit their expression of emotions, and secondly the suspension of identity is part of maintaining professional boundaries. Music was considered as a strategy for enabling staff to process experiences and as a common ground with parents that provided shared experience but acknowledged difference in identities. This was supported by the theory of ‘mattering’ (59). It is proposed that if parent and staff identities were acknowledged this may increase a sense of mattering to one another by feeling seen and valued and therefore increase psychological safety.

Additionally, the impact of social and historical trauma such as racism and stereotyping on health inequality and staff burnout (60, 61) could not be overlooked. Participants reported experiences of oppression and discrimination due to intersects of their identities. These experiences had led to the sense that expression of identity was challenging and in turn this limited parents’ opportunities to connect their infant with their cultural identity. Recent approaches to acknowledging cultural identity, promote a stance of cultural humility to take an enquiring approach to cultural values. This co-design team felt that this approach was vital for the intervention. Firstly, acknowledging and valuing identities by celebrating and promoting their inclusion in sounds and songs, which could create an open conversation between parents and staff and create acceptance of difference.

Theme 2: Trauma

Ways to support parents and staff to draw upon safe coping strategies became a focus when considering the multiple areas of trauma that parents and staff experienced on the unit and their avoidance to engage in processing their emotions due to potentially becoming overwhelmed. There are trauma informed frameworks that aim to build safety to address state regulation (62). Therefore, the co-design team felt that music could enable parents and staff to feel in control of their body’s response and able to recover, moving safely between activated and safe states. They considered examples of using a trauma informed framework (63) to create approaches which promote a feeling of safety in connection to the use of music. In the transcripts it could be noted that many parents already used music to support them navigate through periods of high stress. Therefore, it seemed appropriate to continue to encourage and support this use of music.

Theme 3: Impact of the staff-parent relationship

Reported experiences of participants suggest that if neonatal staff are unable to regulate parent state on the unit and parents become isolated from their usual support networks, then parents become disconnected from their body’s experience and can remain in a perpetual state of threat and trauma. Research has recognised that repeated incidents of trauma without coregulation in an individual’s environment or from outside sources of support results in a desensitisation of the autonomic nervous system which if not interrupted can lead to a cycle of dysregulation (64). Once in a cycle of dysregulation parents who are not supported are likely to react to perceived danger/threat. Similarly, if staff are not supported and maintain an activated state of self-protection, they are likely to either appear withdrawn or authoritarian. From participant experiences it was clear that reactions stemming from a heightened state of arousal because of unconscious attempts to maintain survival, have significant impact on both staff and parents and can lead to a breakdown in relationships. Much of self-regulation relies on others, so the lack of social connections perpetuates stress and loss of safety resulting in the trauma effecting the entire system: staff, parent, infant and external relationships (25). With the clear impact of relationships on parental and staff experience and the known value of social relationships on regulation, strengthening social connections became a primary area of focus for the intervention. On the neonatal unit these social connections were considered not only between parent and staff but also across the staff team. On reflection of staff’s acknowledgement of the positive impact of a supportive unit culture on their capacity for parents, the use of music to strengthen staff team relationships was considered.

### 4.3 Development of an intervention logic model

**Figure 5:**
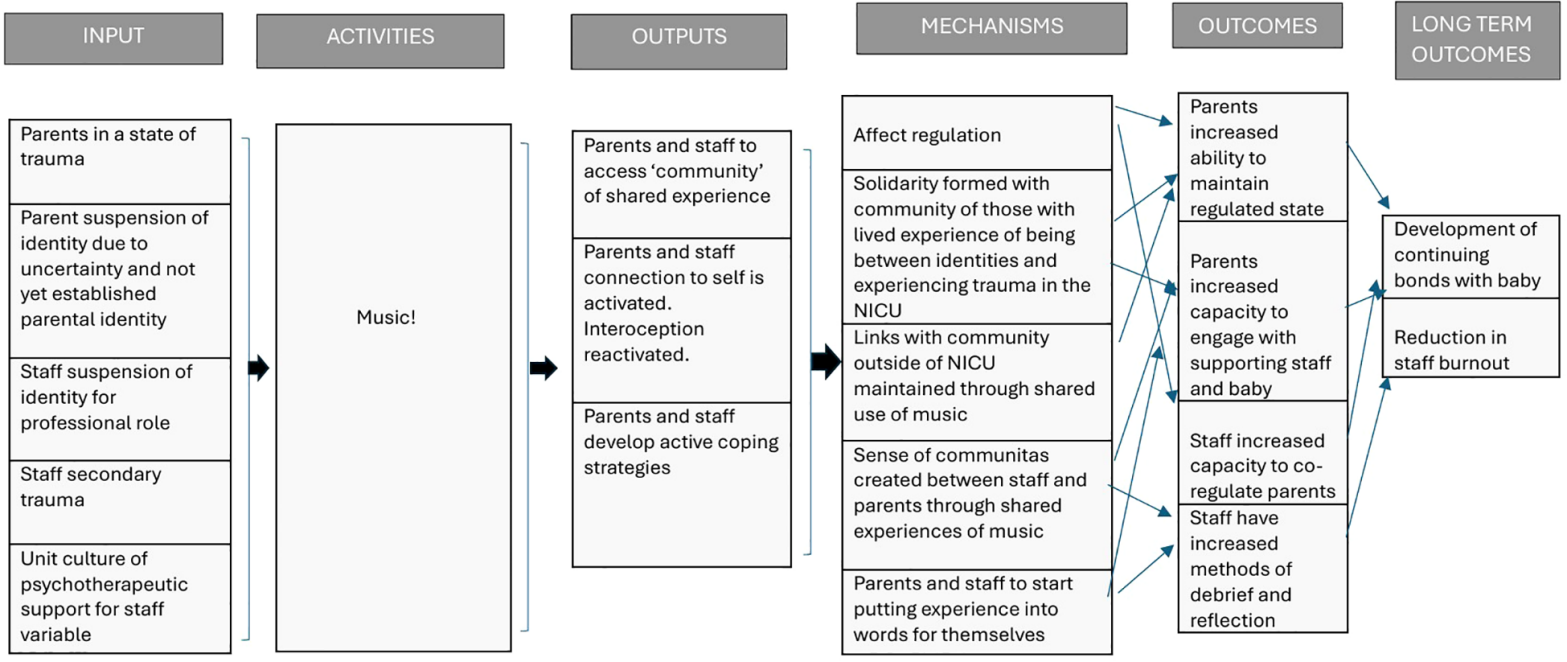
Logic model of the music therapy intervention.

Once the above three themes had been identified and linked to theory, a logic model (**Figure 5**) was created by the lead researcher and reviewed by the wider research team. Informed by the themes and theory, the intention of the intervention was to support parents and staff in maintaining a state of psychological safety through increasing their capability to regulate while on the neonatal unit. Increasing their capability to regulate would be achieved through supporting social relationships and connections and reestablishing connections to a sense of self and internal experiences. It was hoped that, in turn, this would: a) increase parent’s capacity to engage with their infants b) increase parents’ capacity to engage with support services which would strengthen their self-efficacy in caring for their infants; c) increase staff capacity to support parents; and d) enable parents and staff to be in a psychological state where they feel able access further psychological support should they want to.

A further two co-design meetings (meetings 3 and 4) were then held for ideation (generation of ideas and exploration of solutions), prototyping (modelling of a concept) and graphic design.

## 5 The ‘develop’ phase

### 5.1 Co-design meeting 3 (the develop phase)

At the beginning of meeting three, the lead researcher presented the findings from the analysis processes and explained the theories of trauma and identity liminality which were being used to inform the process and mechanisms of change of the intervention. The logic model was presented for the co-design participants to review and then used to generate ideas for the active components of the intervention. They were reminded to consider their ideas for the intervention within a wider context of uncertainty and traumatic stress, as well as acknowledging on-going oppression, historical trauma and power-privilege dynamics that parents and staff experience daily. When generating their ideas, the co-design participants were asked to consider:

- what a MT intervention should look like.
- what resources could be created to help parents and staff draw on the supportive elements of music that were highlighted by participants.
- how music may be used to support parents and staff through traumatic events and periods of transition of identity.

Co-design members were each asked to propose four ideas for an intervention that would support their peers and future parents through the neonatal experiences. They were asked to think about how best to reconnect staff and parents to their sense of self and identity and help them draw on their previous strengths. Based on the logic model, the group proposed that the intervention should aim to:

- Support parent and staff affect regulation.
- Create solidarity with others with similar lived experiences.
- Link parents and staff to their community/network outside of the NNU through shared music.
- Develop sense of ‘communitas’ between staff and parents through music.
- Support staff and parents to start putting experiences into words for themselves. For example, recognising their emotions, physical symptoms, and thoughts.

The co-design participants then either sketched or described their ideas on an A4 paged divided into four sections. The lead researcher provided a short timeframe for this initial ideation with a suggestion that these initial ideas should be unlimited by budgetary considerations to enable imagination without constraint. The unlimited budget aimed to promote creative thinking outside of their own experiences but in response to the findings from the data. Each member then presented their initial ideas to the group. The ideas were then refined in the second part of this meeting to ensure that they were able to deliver the outputs required to produce the outcomes in the logic model and the viability of ideas considered. After presenting their ideas, the participants reflected on commonalities within their ideas and what elements they would like to prioritise to take forward into the next design phase.

### 5.2 Findings from co-design meeting 3

Examples of ideas generated by parents and staff based on the logic model and the final ideas prioritised can be found in **Tables 2** and **3**. The viability of the ideas was also considered and refined to ensure the intervention was feasible within the current hospital environment and setting. For example, on further reflection of staff engagement with therapeutic support, it was felt that although support for staff could be offered, at times of high stress, staff would rarely engage with it. Therefore, support for staff needed to be something flexible which could be used individually but still promote connection across the team. The final concepts taken forwards for prototyping comprised the following components:

- Support from a music therapist to create ‘musical gifts’ (see **Table 2**) for the infant from their parents and wider support networks. The aim of this component is to strengthen social connections external to the unit. The intention is to support celebration and connection of both parent and infant to their cultural identity, connection both to their wider community of support.
- A blog of veteran parent and staff experiences of using music to support themselves on the neonatal unit with the aim of reducing the isolation that comes with the experience of trauma. The intention is to encourage NNU parents and staff to consider strengths they have previously found in sounds and songs and enable them to start considering what music they may use on the unit.
- A diary for parents that provides prompts for musical ideas and encourages body awareness. By increasing body awareness, the intervention aims to reestablish parental interoception which is often lost in an environment in which our mind perceives we are under threat. The intention is that by understanding their body’s response to their experience, parents could use music to help regulate to a safe state physically and emotionally.
- The development of shared unit playlists for staff to develop social connections within the staff team. This component aims to celebrate staff identities across the staff team and boost a sense of community support between staff. The intention is that by increasing the sense of a supportive environment and ideas for using music to support psychological regulation, staff burnout could be reduced which would increase their availability for supporting parents.
- Staff to wear ‘Ask me’ badges as a signal of a safe relationship opportunity in the sharing of music between parents and staff. These badges are to act as an invitation for a non-medical conversation that would acknowledge and celebrate identities. This could increase parent feelings of being valued as they are, rather than having to adapt to a western-idealised model of support. Additionally, the intention is that increasing non-medical conversations between staff and parents may help them to better relate to each other at times when medical decisions and cultural beliefs conflict.

**Table 2:** Examples of ideas and initial prototypes for parents.

Aim	Idea	Prototype taken to final meeting
Support parent regulation	Music Therapist creates lullaby/calming version of parent’s chosen sound or song. It is played live in a separate ‘music’ space and a recording is given to parents and staff to play through their phones and through headphones at bedside. Two sets of headphones each to share experience and played through the incubator.	A diary providing ideas for creating sharing music and optional in person engagement. Recordings shared either by parent singing or humming or played through incubator speakers.
Solidarity with others with lived experience	Online digital platform to allow people in the same situations/difficult situations to gather ideas and continue them as they fit their situation.	Parent and staff blog of experiences using music
Link to community off the NICU	The hub (app) will send family ideas on how to create content for the baby. This is written in first person as if it is a message from the baby to make them more real to the family, “Read me a short story or sing me a song’	‘Musical gift’: Music therapist connects with family/community to support them in creating a ‘musical gift’ for baby.

**Table 3:** Examples of ideas for staff.

Aim	Idea	Prototype taken to final meeting
Support staff regulation	Protected staff music therapy time once a week. Staff engagement- ask staff about their music preferences	Create a unit staff playlist. Playlist advertised on unit. Staff encouraged to add their music to unit playlists
Supporting staff and parent relationship	Parents share sounds/songs with staff so staff can build a more human/less medical picture of culture/passions/personalities of family	Staff wear “Ask me” badge to encourage open conversation about music
Support staff regulation	Monthly team music nights, share food, listen to music, and connect (you are only as good as your team)	Shared unit playlists for social community on unit

**Figure 6:**
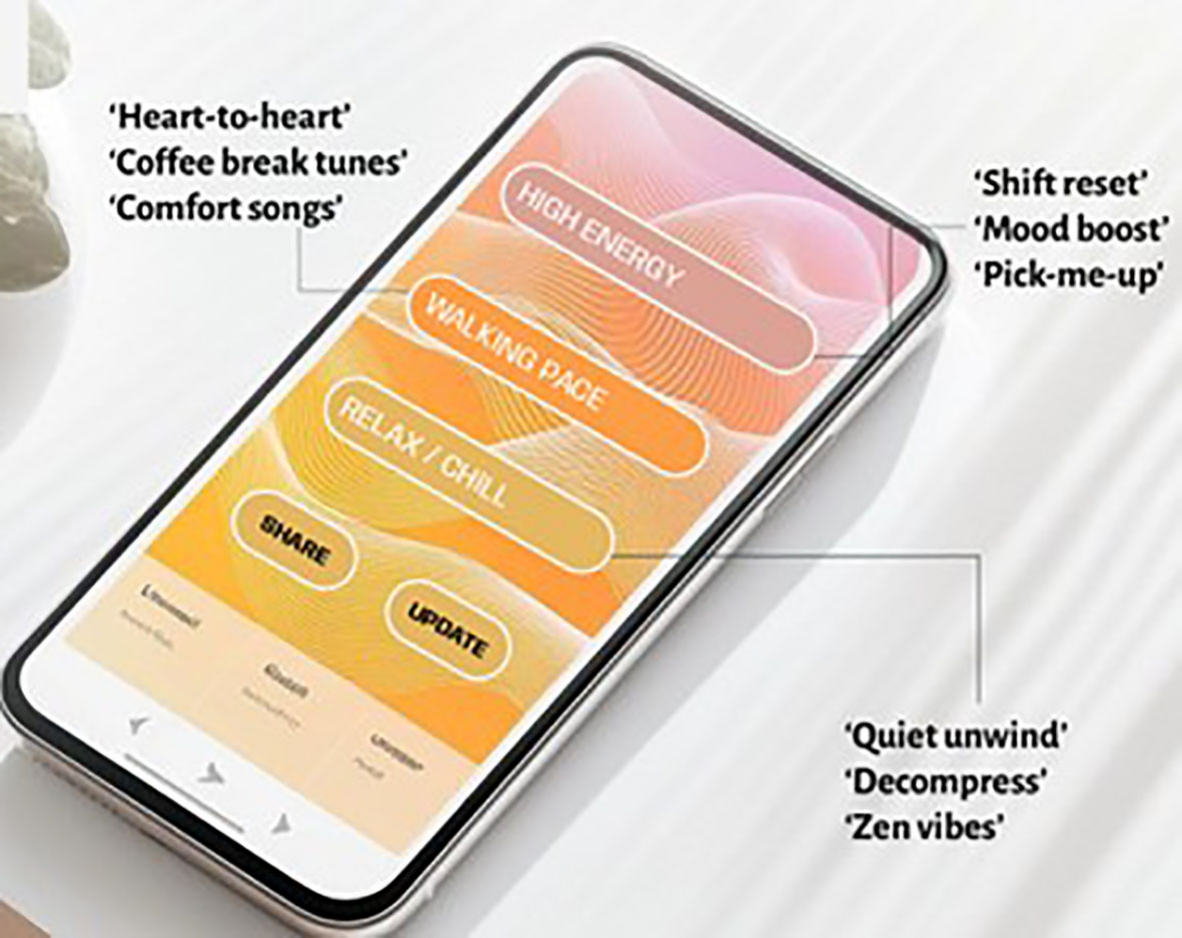
Playlist program prototype.

**Figure 7:**
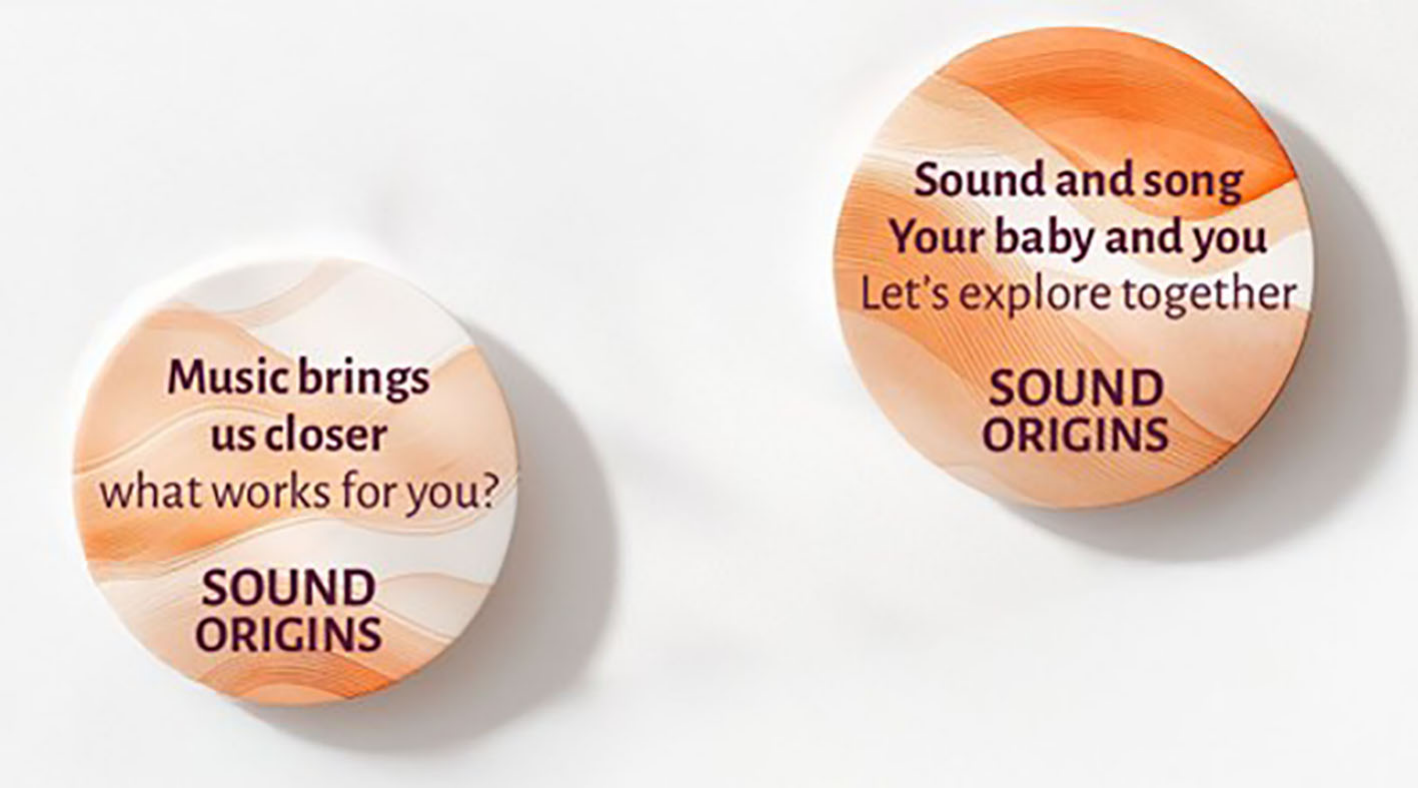
‘Ask me’ badge prototypes.

From the conversations and ideas in meeting three, the lead researcher proposed the ideas to a professional design team specialising in graphic communication and technology for further development. These designers had not been part of the analysis process and work directly in response to the findings and outcomes from the co-design meetings. They created a digital concept of the intervention with the five components to share at the final co-design meeting for refinement in March 2025 (visual examples of concepts are provided in **Figures 6**, **7**).

### 5.3 Methods for co-design meeting 4 (prototyping)

At the final co-design meeting, the team reviewed the components of the intervention. This included digital visualisation of a blog, diary, playlist app and webpage. This meeting was held online and summarised each stage of the co-design process before presenting each component. In between each component, the co-design members were invited to comment on the prototypes.

After the meeting, the lead research and designer considered all the suggestions and recommendations to refine the intervention. A further newsletter was produced to share the final intervention concepts and the earlier qualitative phases of the wider study to invite further comments. Using the newsletter, social media presence and the charity groups involved, parents with lived experience were asked to send their music to the lead researcher for the development of the playlists for future parents on the neonatal unit.

#### 5.3.1 Findings from co-design meeting 4

Participants reflected that the blog would be better presented as a parent playlist with a separate page to present the reasons for parents’ use of these tracks. Parents in the co-design group felt that a blog may be too overwhelming to read. They felt however that the ability to listen to musical suggestions would be helpful, and at the right time, so would the option to explore reasons behind the suggestions. These reasons would be particularly useful to create a feeling of not being the only parent experiencing being on the NNU with a seriously unwell infant. To increase the accessibility of the new parent playlist and the staff playlists (see image 3), the team suggested that headings for each playlist were either emotion labels or sensory based rather than speed of track (as shown in image) as would be easier to relate to and more culturally transferrable. Additionally, to ensure the playlists could be accessible, it was noted that a device on the unit would need to be provided for those would did not access the internet or digital devices at home. The team also identified that a logic system that would be required to enable music to be filtered (e.g. voice or instrumental, male or female, or pace of music) and should be added to the playlists to enable parents to hear or not hear certain styles of music or sounds.

All team members liked the idea of a diary and badges. They considered the wording for both components. On the staff badges (see image 4) they noted the importance of simple language in a parents first language with the support of a visual icon to assist and encourage communication. The diary was renamed ‘a journal’ as parents felt this would be perceived as more valuable and something that might be a keepsake. The content of the journal was refined to create a guide for the use of music and steps for self-awareness, as well as providing space to reflect. Additionally, it was suggested that the link for the website and playlist should be in the journal.

When considering when to have this intervention available, all co-design members felt that this was valuable from the start of admission and could potentially be introduced to parents who were admitted to hospital antenatally with a known diagnosis of uncertainty for the infant’s future. The team reflected that the initial meeting with a music therapist would need to be brief due to the level of involvement of other healthcare professionals.

## 6 Discussion

This co-design process emphasised early on the necessity for MT in the NNU to take a trauma informed approach. While other MT approaches consider the value in assisting the reduction of stress and anxiety for parents and their infants (65–67) there is often little time prioritised on creating a sense of security within the parent and maintaining social connection as part of a trauma informed approach. The ‘Rhythm, Breath, Lullaby’ approach describes the importance of creating a sense of safety in assessment and sessions but does not focus on continuing to strengthen parents’ skills in continuing this without the support of a music therapist (68). Some studies have highlighted that parental anxiety in participating in MT, but this tends to subside as they see the positive effect on the infant (35). However further consideration for what this means for the accessibility of the intervention has rarely been considered. It is possible that further consideration to ensure parents feel confident in their ability to self-regulate would increase parents’ capacity to engage with interventions that support them to connect and support with their infant during admission.

With an increased understanding of their bodily responses to traumatic events and reconnection to their use of sounds to support them returning to a state of safety, it is likely that parental self-efficacy will be increased. The importance of parental self-efficacy is recognised in the ‘Time Together’ approach (69), which highlights parents being supported to bring to awareness their capabilities, to enable them to continue to engage with their infant without a music therapist present. Approaches which develop skill in maintaining psychological safety through drawing on previous strengths provide a universal level of psychological support which is recommended in guidance from neonatal consultant clinical psychologists for psychological provision across UK neonatal units (70). The development of skills in using music for coping is something that could potentially be developed across multiple professions making it a cost-effective base level of support and complimentary to current psychological provision. This model of support aims to change the experience of potentially traumatic events (71).

This co-designed MT intervention straddles both universal and targeted support. It aims to provide an initial universal support which could benefit all neonatal parents, by increasing self-efficacy and support systems, before later offering further targeted support in using music to assist with parent’s involvement in caring for their infant and parent-infant bonding. Some parents on the NICU may not require assistance for using their sounds after gaining an increased sense of psychological safety and confidence in their capability to overcome adversity. However, for parents who have factors which increase the likelihood of ongoing challenges (e.g. due to ongoing trauma and uncertainty or ongoing mental health challenges) further targeted support to interpret and engage with their infant may be valuable. Having considered their values and strengths found in sounds and song, a therapeutic relationship - which can provide ongoing support for engaging and supporting the infant as well as maintaining parental psychological safety - is likely to be more accessible. Furthermore, the initial relationship and foundation of support has the potential to increase the accessibility of other sources of support available on the unit which may help parents in understanding, engaging and therefore bonding with their infant. Using this approach, music therapists would be able to provide accessible support at all tiers. It is likely that this would positively act towards addressing health inequality through provision of psychological support which has been designed by parents and professionals. Further demonstration of the level of psychological knowledge and skills held by trained music therapists would increase the trust in the profession within the UK neonatal network. It may also support consideration of the approach as a complimentary service that is likely to be more accessible to those who have experienced oppression due to decreased need for verbal narrative and processing.

This intervention acknowledges qualitative data and parents’ request for their infant to be known outside of the NNU. It also recognises the value in parents continuing to feel supported by their social networks. While some approaches use music that relates to family and their identities (29, 72) working with external family and friends to support families on the unit has not previously been considered. It is likely that this component will have significant value for the parental neonatal experience and for increasing wider social understanding of the environment.

### 6.1 Requirements for implementation of the intervention

Due to the complexity of trauma experienced by those on the neonatal unit - and the fragility of many infants on the neonatal unit - it is vital that professionals delivering this intervention have a high level of psychological training and neonatal behaviour observation knowledge and skills. Currently, there is no standardised requirements for music therapists working on neonatal units in the UK but findings from this study - which further reinforce the psychological impact of neonatal admission - suggest a need for additional training within the current Music Therapy Master’s degree. This was further evidenced by the systematic review which demonstrated value in professionals delivering interventions to be knowledgeable of the environment and infant development (51). To support healthcare professionals to engage with the intervention, effective communication of how it was co-designed and the value of their involvement is important. Animations have been developed and are available on the project website to support this (see ‘Resources’).

When implementing the intervention, it is recommended that the use of sound and song on the NICU with the infant is monitored to ensure the safety of the infant and minimise any negative effects on parents of other infants in close proximity. A guide to the use of music has been created to support this on the website. The promotion of live, responsive interaction using sound is vital to ensure that this supports infant development (73, 74). An appropriately trained music therapist can support parents in noticing infant cues and increase parents’ confidence in adjusting sounds accordingly.

### 6.2 Potential for use beyond the UK neonatal unit

This intervention does not rely on provision of a separate space for time with the music therapist or the intervention components. Components have been designed to be able to be engaged with whilst requiring only minimal support from professionals or additional instruments and devices. The resources promote the use of individual sounds and songs and the use of voice for interaction and can be accessed via the website, and are available in Arabic, English, Romanian, Somali and Turkish to increase accessibility. The resources are designed as a starting point for connection and therefore can be further built upon after initial engagement in different environments. Although this study focused on supporting the parent-infant bond on the neonatal unit, it is possible that a similar approach could be used in other environments to support parent-infant relationships. To ensure and promote the safe interpretation of infant cues and adjustment of sound in response to these, we advise that the intervention is delivered by a music therapist and that specialist training is undertaken if providing the intervention in settings with infants with complex care requirements or prematurity. Importantly, the consideration for the psychological wellbeing of staff supporting parents and infants should not be overlooked.

## 7 Plans for testing

The research team plan to test the acceptability of this MT intervention within the NICU of a hospital in the UK. Testing will aim to engage 12 parents of 12 infants with an uncertain future on admission to the neonatal unit. The researchers aim to ensure a variety of intersects of identities is held across the parent participants with no more than 2 holding the same intersects of identities to help evaluate its acceptability. All parents will be asked to complete an acceptability questionnaire and invited to interview to discuss their experiences and use of the components further. Nursing staff supporting these families will be provided with badges to wear and encouraged to develop shared nursing team playlists. The frequency of their use will be assessed and a focus group held to further discuss the acceptability of the components of the intervention for nursing staff. Although the intervention is likely to be valuable across the neonatal population, those experiencing uncertain futures will be prioritised to assess acceptability for those who might be excluded from other sources of support due to fear from professionals of overloading or impacting an already limited precious family time. Feasibility of the intervention will be indicated by high engagement with the intervention components in combination with minimal burden or cost to personal and family lives or conflicts with individual’s value and belief systems.

## 8 Implication on future practice and research

This is the first MT approach to consider strengthening social connections external to the unit as the initial aim and promote the strengths of those experiencing trauma to assist them to achieve a state of psychological safety from which they are able to access further psychological support and assistance in engaging with their infant. Further research on the development and accessibility of psychotherapeutic support for HCPs on neonatal units would also be beneficial. Specifically, new interventions should aim to reduce burnout and increase staff capacity to be available for parents. Developing interventions which address the many layers of trauma within neonatal care for infant, parent, and staff - and further consideration of their impact on one another – would also improve the experience for everyone within the NNU environment. Additionally, further research on increasing awareness of the neonatal experience to improve community support for families on neonatal units may improve parent and staff experiences as well as reduce symptoms of trauma. Further exploration of using a similar MT approach to support parents with an antenatal diagnosis would also be valuable.

The individualised focus and culturally sensitive nature of this intervention increase the likelihood that this intervention may be transferable globally. Further testing on a wider scale would support the understanding of the interventions’ transferability outside of the UK. Additionally, testing of the interventions in settings other than the NICU would further increase the transferability of the intervention to other settings where the parent-infant relationship requires support due to increased levels of uncertainty and trauma.

## 9 Limitations

Professionals and parents within the co-design group were all enthusiastic about the potential of MT to support parents prior to the study with many experiencing MT with the lead researcher. This resulted in many members holding a prior belief that MT could support future parents. Every effort was made to ensure that the study remained grounded in participant voices. These were predominantly from parents who had not experienced MT and reflected the needs of parents and staff more broadly. However, participants who engaged in the co-design team were unlikely to conclude that music did not have the potential to support parents and infants. Additionally, the lead researcher who provided their music therapy sessions often used ‘songs of kin’ (29) in their practice and therefore parents in the co-design team were likely to feel that this was an essential component of music therapy. This may have influenced their decision to have a similar component as part of the intervention. Finally, this intervention was designed by parents and healthcare professionals with experience on UK neonatal units and palliative care services (except for one parent who had experience of Asian Pakistani neonatal care in addition to UK neonatal care). This has resulted in the intervention being tailored to the NHS and the NICU environment in the UK. Whilst the researchers hope that this will be a transferrable intervention internationally, further testing is required.

## 10 Conclusion

This study adds to current research that demonstrates the value of co-design methods to develop healthcare services. This collaboration with parents and professionals with lived experiences has created an intervention that is unlike any other music therapy approach currently available. It is the first co-designed, theory-informed neonatal music therapy intervention and the first neonatal MT therapy study to prioritise the experiences of parents with infants with uncertain futures increasing the accessibility to the neonatal parent population. The intervention has the potential to enhance current psychological support for both parents and staff which is accessible due to cultural, linguistic, and religious appropriateness. This in turn could begin to address some of the health inequality currently present in psychotherapeutic support and neonatal care.

## 11 Resources

Links to the webpage and resources developed as part of this study can be found at www.soundorigins.org. The researchers would like to share these resources but would like to request that anyone intending to use these resources please contact the lead researcher before doing so.

## Data Availability

The original contributions presented in the study are included in the article/**Supplementary Material**. Further inquiries can be directed to the corresponding author.
