# Supplementary material for: Supporting continuing bonds for parents with infants with uncertain futures on neonatal units in the United Kingdom: co-designing a culturally sensitive music therapy intervention
Source: Front Psychiatry. 2025 Jul 21;16:1633878. doi: 10.3389/fpsyt.2025.1633878 (PMC12320057; doi:10.3389/fpsyt.2025.1633878)
Supplement: Supplementary file 1 [file Table1.docx]

Appendix 1: Code Tree

*Primary themes*

| IDENTITY | Lost identity | Lost identity |
| --- | --- | --- |
|  |  | Feeling alone |
|  |  |  |
|  | Parental identity on the NICU | Moments of feeling like a parent |
|  |  | Needing permission |
|  |  | Feeling baby isn’t 100% yours |
|  |  | No protected time as parent and baby |
|  |  |  |
|  | Business parent | Agreeing to medical decisions and keepsakes |
|  |  | Stay focused on practical tasks and positivity rather than emotions |
|  |  | Confidence as the weeks go on |
|  |  | Avoiding being an emotional burden for partner |
|  |  |  |
|  | Stuck in identity transition | Feeling trapped |
|  |  | Lack of hope |
|  |  | Feeling like a failure/ low self confidence |
|  |  | Helplessness |
|  |  |  |
|  | Religion and Culture | Support from religion/ Not feeling alone. |
|  |  | Celebration of parental and family identity |
|  |  | Guilt for being unhappy with god’s choices |
|  |  |  |
|  | Baby identity | Baby a human not a diagnosis |
|  |  | Awareness of difference between baby and others |
|  |  |  |
|  | Parent preference for male staff | Preference for male staff |
|  |  |  |
|  | Expectations of a professional | Expectation not to show emotion |
|  |  | Giving everything you have |
|  |  |  |
| TRAUMA | Staff responsibility | Responsibility for the outcome of the baby |
|  |  | Being a vessel of parents hate, anger and grief |
|  |  |  |
|  | Partner experience | Feeling secondary to mothers |
|  |  | Concern for birth parent wellbeing/ watching distress |
|  |  |  |
|  | Secondary trauma | Secondary trauma |
|  |  |  |
|  | Emotional resilience (staff) | Emotional turmoil |
|  |  | Moral turmoil |
|  |  | Feeling helpless |
|  |  | Becoming emotionally hardened |
|  |  | Years of experience making end of life easier |
|  |  |  |
|  | Staff support | Taking on the role of counsellor but not trained as one |
|  |  | Emotional burden taken home |
|  |  | Lack of psychological support for staff |
|  |  | Psychotherapists unapproachable for staff |
|  |  | Supportive staff culture |
|  |  | Younger generations more likely to request support |
|  |  | No training for end of life |
|  |  | Multiple sources of support for parents reduces staff burden |
|  |  |  |
|  | Fear and shock | Lack of time to process |
|  |  | Auditory truama |
|  |  | Fear of loss and causing harm |
|  |  | Uncertainty for the future |
|  |  | Unexpected outcomes/ change to future life |
|  |  |  |
|  | Staff difficulty with loss | Only seeing baby at their sickest |
|  |  | Difficult when parents don’t visit |
|  |  | Challenges with loss when bond is made |
|  |  | Distancing in order to protect in anticipation of loss |
|  |  |  |
| HCP- PARENT RELATIONSHIP | Religion and culture | Cultural acknowledgment |
|  |  | Oppression due to identity |
|  |  | Sometimes you’re not the right person to support |
|  |  | Cultural difference is challenging |
|  |  | Lack of appropriate talking therapy |
|  |  |  |
|  | Professional communication | Difference in communication between teams |
|  |  | Mediating people who create a bridge |
|  |  |  |
|  | Staff understanding and communication | Constant negative words and feedback |
|  |  | Making assumptions about parent capabilities |
|  |  | Not being listened to leading to lack of trust |
|  |  | Staff not showing an interest in parent or baby |
|  |  |  |
|  | Supportive staff | Belief that staff have done everything they can |
|  |  | Staff being supportive |
|  |  |  |
|  | Somone who understands | Having someone who understands |
|  |  | Reassurance and encouragement from staff |
|  |  | Religious staff support for parents |
|  |  |  |
|  | Communication with family | Breakdown in relationships with families |
|  |  | Creating bonds with families |
|  |  | High turnover of babies |
|  |  | Lack of trust from parents |
|  |  |  |
|  | Press and social media | The negative impact of press on parents |
|  |  | Staff have no right to reply |
|  |  |  |

*Secondary themes:*

| Judgement | Judgement and scrutiny from parents |
| --- | --- |
|  | Judgement from staff |
|  |  |
| Moments of hope | Moments of hope and dreaming |
|  | Seeing positive changes and milestones |
|  | Family time |
|  |  |
| Comfort | Items that bring baby comfort |
|  |  |
| Choice | Lack of choice |
|  |  |
| Connection | Moments of connection |
|  | Continuing to connect post-death |
|  |  |
| Separation | Leaving baby each day to go home |
|  | Separation from baby post death |
|  |  |
| Transitions | Difference between units |
|  |  |
| After NICU | Life after NICU harder |
|  | Wanting to forget NICU |
|  | Regrets for not doing more |
|  | Staff don’t get to see what happens after NICU |
